# Possible causal relationships between competitive swimming in growing age and three-dimensional dentoalveolar development

**DOI:** 10.1186/2196-1042-14-17

**Published:** 2013-07-26

**Authors:** Armando Silvestrini-Biavati, Claudia Capurro, Alessandro Ugolini, Andrea Carlo Butti, Antonino Salvato

**Affiliations:** Department of Orthodontics, University of Genoa, Genoa, Italy; Rapallo, Italy; Department of Surgical, Reconstructive and Diagnostic Science, Oral Rehabilitation Unit, University of Milan, Milan, Italy; Department of Orthodontics, University of Milan, Milan, Italy

## Abstract

**Background:**

The aim of this study was to investigate possible links between competitive swimming during the growth phase and the development of the dentoalveolar arches.

**Methods:**

The study sample included 100 swimmers and a control group of 100 age-matched non-swimmers who had never practised swimming or related sports. Subjects who had had previous orthodontic treatment were excluded. Overjet, overbite, sagittal and transverse parameters, arch dimension, crowding and oral habits were recorded.

**Results:**

In the swimmers, there was a significantly higher frequency of molar symmetry (*P* = 0.04), together with a greater number of Class I subjects. The overjet in the swimmers was mainly normal, but the arch dimensions were significantly wider (+10% in the upper arch; *P* < 0.001). Similarly, the swimmers showed significantly less severe crowding (*P* < 0.001) and significantly reduced oral habits (*P* < 0.001).

**Conclusions:**

Our data and analysis demonstrate that competitive swimming during the growth phase has a favourable effect on dental arch development in the sagittal, vertical and transverse planes.

## Background

Swimming is recommended in children to promote harmonic development in all of the body districts. It also involves respiratory training, in which deep inspirations are followed by long expirations that are controlled by the lips. These actions might have positive effects on maxillary development, according to the functional matrix theory [1, 2]. Indeed, the upper dental arch and palate (as anatomical structures that are at the same time the oral cavity roof and nasal cavity floor) can be influenced by breathing and tongue action during growth. Vargervik et al. [3] clearly demonstrated a direct relationship between oral breathing and maxillary shape modifications in the rhesus monkey, especially for the upper jaw and the anterior upper teeth. Several studies have also shown the contributions of the tongue to morphological maxillary growth [4–6], whereby when the tongue posture and action are not correct, this can provide the origin of dentoalveolar modifications [7–10].

In the present study, we investigated possible links between competitive swimming sports (i.e. swimming, water polo) and the development of the dentoalveolar arches. A previous study reported that specific physical training practised regularly can act as a positive factor in dentoalveolar growth [11]. Therefore, according to these concepts, in the present study, we recorded three-dimensional dental arch views, first molar relationships, overjet, overbite, transverse parameters, arch dimension, crowding and oral habits in swimmers, as those who had practised competitive swimming sports intensively for at least six consecutive years during growth, versus non-swimmers, as those who had never practised swimming or related sports.

## Methods

The study protocol was approved by the scientific Committee of the Department of Biophysics, Medicine and Dentistry, University of Genoa, Italy, and it was carried out in accordance with the ethical standards.

Written informed consent for publication of this report and any accompanying images were obtained from all the patients before their enrolment in this study.

### Subjects

A group of subjects was identified and defined as the study group of ‘swimmers’. These swimmers included 100 subjects (62 men and 38 women) who were selected from among athletes in the Genoa area (northern Italy) and who were aged between 16 and 30 years (mean age, 23 ± 3.8 years). As swimmers, they had participated in regular and intensive swimming training for 8 to 12 h per week, which had continued for at least six consecutive years during their growth phase (from 4 to 10 years old). Subjects who had had previous orthodontic treatment were excluded.

A second group of age-matched subjects was identified and defined as the control group of ‘non-swimmers’. These non-swimmers included 100 subjects (62 men and 38 women) who were randomly selected from among economics and dentistry students at Genoa University and who were aged between 18 and 30 years old (mean age, 22.6 ± 3.8 years). The subjects in this control ‘non-swimmers’ group had not had orthodontic treatment and were not competitive swimmers.

### Dental examinations

A dental examination was performed with each subject, and a brief case history was compiled, in which their oral habits (breathing type, swallowing pattern, lip seal) were recorded, together with their training frequency and duration. Dental casts were obtained, and the sagittal and transverse relationships, overbite, overjet, arch dimension and crowding were evaluated.

Normal overjet was defined as between 0 and 2 mm (depending on tooth thickness), and we considered good overjet to range from 0.5 to 2 mm. In the vertical plane, the overbite was considered correct when the upper incisors covered the lower incisors by about 1.5 to 2.5 mm. In the transverse plane, the buccal cusps of the upper teeth were correctly positioned if they were outside the lower buccal cusps on both sides. The occlusal interpremolar and intermolar widths were measured with a digital gauge (DCA150B Velleman, Gavere, Belgium), respectively, as the distance of the palatal cusps of the first bicuspids and as the distance between the mesiolingual cusp tips of the second permanent molars. The arch dimensions were calculated using the appropriate templates based on the Ricketts pentamorphic arches, as suitably increased (+5%, +10%, +15%) or reduced (−5%, −10%, −15%), starting from the original image [12]. In this way, we had six templates for each shape, which were laid on the dental casts until the best match was found. This analysis was performed by two operators (ASB, AU) at different times; when these operators did not agree, a third examination was performed by a third operator (ACB).

For the space analysis, each single tooth was measured with the digital gauge (in millimetres), obtaining the necessary space needed for aligning these teeth in the arch A. The real length of the arch was calculated using a brass wire that was modelled on the buccal cusp of the dental arch B. The difference of A minus B showed a lack of space if negative or an excess of space if positive.

### Method error

To standardise the measurements, all of the data were collected by the principal investigator (C.C.) and checked by another senior clinician (A.S.B.). The measurements were repeated on 10 randomly selected casts to determine the error of the method between the first and second measures. Intraclass correlation coefficients were calculated to compare the within-subjects variability to the between-subjects variability; all of these values were greater than 0.90. The standard deviations between repeated measurements were in the range of 0.06 to 0.17 mm for all of the measurements (mean variation, 0.1 mm). Overall, the method error was considered negligible.

### Statistical analysis

The sample size was calculated *a priori* to obtain a statistical power of the study greater than 0.90 at an alpha of 0.05. Means and standard deviations were computed for each group for overbite, overjet, sagittal and transverse relationships, arch dimension and crowding. The Shapiro-Wilk's test demonstrated the normal distribution of data, and thus, the swimmer values were compared with data from the control, non-swimmer, group by parametric *t* tests for independent samples and chi squared analysis. The level for statistical significance was set at *P* < 0.05.

## Results

Fifty-two swimmers had an Angle's Class I molar relationship, as compared to 37 non-swimmers (Table 1). Although this difference was not significant, the 70 swimmers with a symmetric molar relationship (left and right sides in the same subject) was significantly greater compared to the 55 non-swimmers (Table 1; *P* = 0.04).

**Table 1:** **Angle’s Class bilateral molar relationships, symmetry of the molar relationship and crossbite**

Variable	Swimmers	Non-swimmers	***P*** (chi squared)
**Bilateral molar relationship** ^**a**^			
Angle's Class I	52	37	NS
Angle's Class II	14	12	NS
Angle's Class III	4	6	NS
**Right/left molar relationship**			
Same	69	55	0.04
Different	31	45	
**Crossbite**			
Present	8	12	NS
Absent	92	88	

^a^Considering only those subjects with the same molar relationships (swimmers, *n* = 70; non-swimmers, *n* = 55). NS, not significantly different.

The swimmers showed greater overbite than the non-swimmers (2.9 ± 1.6 vs. 2.6 ± 1.5 mm), but this finding was not significant.

The mean values for the overjet did not show any significant differences between the swimmers and non-swimmers. However, if we consider an overjet of 3.5 mm as close to normal [13], there was a trend for a greater number of swimmers (70%) with a 1.5- to 3.5-mm overjet than seen for the non-swimmers (58%; chi squared, *P* = 0.08). Alternatively, 10 out of 100 swimmers (10%) had an overjet greater than 4 mm compared to 12% of the non-swimmers (chi squared, *P* = 0.6).

In the transverse plane, there were only eight cases of crossbite (8%) for the swimmers, which was not significantly different from the 12% in the non-swimmers (Table 1).

In the analysis of the transverse interpremolar and intermolar arch widths, these were all comparable between the swimmers and non-swimmers (Table 2).

**Table 2:** **Transverse interpremolar and intermolar widths**

Variable	Swimmers	Non-swimmers	***P*** (Student's ***t*** test)
**Interpremolar width**			
Mean ± SD (mm)	30.7 ± 2.1	30.2 ± 3.0	NS
Range (mm)	26 to 37	23 to 36	
**Intermolar width**			
Mean ± SD (mm)	47.9 ± 3.6	47.4 ± 3.6	NS
Range (mm)	40 to 59	38 to 55	

SD, standard deviation; NS, not significantly different.

For the arch dimensions, as given in Table 3, with the upper arch, the data showed an increasing shift for the swimmers, with significance for the 5% reduction for the non-swimmers (*P* = 0.001) that was accompanied by significance for the 10% increase for the swimmers (*P* = 0.001). This was similar for the lower arch, with the significance of the 5% reduction for the non-swimmers (*P* = 0.001) accompanied by significance for both no change (*P* = 0.001) and for the +15% increase (*P* = 0.05) for the swimmers.

**Table 3:** **Upper and lower dental arch dimensions, according to the appropriate templates based on the Ricketts pentamorphic arches, and dental crowding**

Variable	Swimmers	Non-swimmers	***P*** (chi squared)
**Upper arch dimensions (%)**			
−15	0	0	NS
−10	4	4	NS
−5	6	24	0.001
0	36	42	NS
5	0	0	NS
10	45	27	0.001
15	9	3	NS
**Upper arch crowding (mm)**			
>−3.5	2	15	0.001
−3.0 to −0.5	49	41	NS
0	35	30	NS
0.5 to 3.0	12	14	NS
>3.5	2	0	NS
**Lower arch dimensions (%)**			
−15	0	0	NS
−10	4	4	NS
−5	7	24	0.001
0	37	41	NS
5	0	0	NS
10	43	27	0.01
15	9	3	0.048
**Lower arch crowding (mm)**			
>−3.5	8	22	0.005
−3.0 to −0.5	54	53	NS
0	27	18	NS
0.5 to 3.0	11	7	NS
>3.5	0	0	NS

Negative, reduced dimensions/lack of space; positive, increased dimensions/excess of space.

NS, not significantly different.

These findings were confirmed by the significantly correlated within-subject comparisons of the dimensions of the upper and lower arches; in the swimmers, more of the subjects (96%) had both arches increased or reduced in the same way, as compared to the non-swimmers (82%; *P* = 0.001). This thus showed a more harmonious development of both of the dental arches in the swimmers.

Table 3 also considers the upper and lower arches with respect to crowding and excess of space. This space analysis showed significantly reduced crowding in both the upper and lower arches for the swimmers, as compared to the non-swimmers (>−3.5 mm: upper, 2 vs. 15, *P* = 0.001; lower, 8 vs. 22, *P* = 0.005).

In the swimmers, the oral habits monitored showed significantly lower prevalence rates when compared to the non-swimmers (Table 4, oral breathing, *P* = 0.001; lip incompetence, *P* = 0.001; abnormal swallowing, *P* = 0.048).

**Table 4:** **The group prevalence rates for the oral habits monitored**

Oral parameter	Swimmers	Non-swimmers	***P*** (chi squared)
**Breathing**			
Nasal	73	45	0.001
Oral	27	55	
**Lips**			
Competent	80	49	0.001
Incompetent	20	51	
**Swallowing**			
Normal	59	45	0.048
Abnormal	41	55	

## Discussion

The assumptions of the present study include the correct neuromuscular function for the development of the dental-maxillary complex and the importance of correct breathing, and normal tongue function and posture (both in swallowing and in the resting position). Alterations of these factors are well known to have roles in the aetiology of malocclusions [1, 3, 5, 6, 8, 14].

Indeed, in a study that looked at the correlations between orofacial muscle activity and craniofacial morphology, Lowe [15] showed that low values for the genioglossus muscle were correlated with an open bite and short total face heights, with low values for the masseter muscle associated with low overbite measurements. He concluded that the interdependence of the tongue and jaw muscle activity and facial morphology suggested a contribution of the musculature to the development of the dentition. Watanabe and Watanabe [16] also investigated muscle activity and its influence on dentofacial morphology in normal adults, and their study concluded that dentofacial morphology is influenced by the jaw-opening and jaw-closing muscle functions.

Ciavarella et al. [17] showed that asymmetric muscle activation may influence maxillary growth in adolescents with unilateral posterior crossbite; Tecco et al. [18] investigated (by surface electromyography) the correlations between neck, trunk, and masticatory muscles with temporomandibular joint disorders.

Several reports in the medical literature have emphasized that in athletes, and particularly in swimmers, related effects are found concerning bone metabolism and muscular-postural activity. In swimmers, Riazanova [19] showed uniform and symmetrical lengthening of the metacarpal bones and thickening of their compact substance due to adaptation to the physical loading. Yamada et al. [20] demonstrated that swimming in rats promotes cartilage formation and bone remodelling. Magkos et al. [21] studied the type and intensity of exercise and the effects on bone mineral density; they reported that sprint athletes have significantly greater bone mineral density, and endurance swimmers have significantly lower bone mineral density than other athletes. In comparing the maximum isometric bite forces of sedentary versus physically trained subjects, Sannajust et al. [22] concluded that the practice of a sport can increase masseter muscle fatigability and will thereby affect posture.

Takeshima et al. [23] showed that swimming produces inspiratory accessory muscle training due to the pressure on the thoracic cage, which results in improved respiratory function; they showed a 7% increase in FEV1 (volume of air expelled in the first second of forced expiration).

The results from the present study need to be carefully considered; we found that compared to non-swimmers, swimmers have wider, more harmonious dental arches, with less crossbite and open bite, and with moderate crowding; this is accompanied by significantly limited incidence of oral habits. A higher percentage of the swimmers showed symmetrical molar Class I relationships, which demonstrates their more symmetrical and correct arch development.

In a study on the prevalence of malocclusion among adolescents in Italy, Ciuffolo et al. [24] calculated a 3.1-mm (±1.8) mean overbite and showed that 41% of the subjects they examined had an overbite greater than 3 mm while 1.7% had anterior open bite. In the present study, similar findings were found in the swimmers who showed a mean overbite of 2.9 ± 1.6 mm, as many as 56.6% had a value greater than 3 mm. Only three of the swimmers had moderate open bite (0 to 2 mm).

Perillo et al. [25], in a survey performed about the prevalence of orthodontic treatment in 12-year-old southern Italian schoolchildren, found an overjet greater than 4 mm in 16.2% while 0.6% had a negative overjet. In the current study, the overjet mean values did not show any significant differences between the swimmers and the non-swimmers; however, the overjet in the swimmers was more localized around the median value (2.5 mm), as indicated by the calculation of the median value deviations in the swimmers (1.0 mm) versus the non-swimmers (1.2 mm). This shows us that more swimmers had close to ideal overjets. Statistical analysis revealed that the weighed mean was 2.6 mm in swimmers, while in non-swimmers it was 2.8 mm; there was also a significantly lower standard deviation in the swimmers (*P* < 0.05).

The greater arch width that was seen for the swimmers represents the most significant of the results in the present study, and particularly for the upper arch. Only 10 swimmers had an upper arch reduction, with 54 with maxillary arch increases. Indeed, the significant correlation of the within-subject comparisons of the dimensions of the upper and lower arches showed more swimmers than non-swimmers with harmonious development of both dentoalveolar arches. Thus, significantly more of the non-swimmers showed differential development between one arch and the antagonist arch, indicating that the swimmers show significantly improved within-subject development of the upper and the lower dentoalveolar arches.

For the space analysis, only a few of the swimmers had severe crowding, particularly in the upper arch where positive tongue actions should promote improved development. Moreover, it must be underlined that the non-swimmers were recruited from among students who had not had previous orthodontic treatment; this would imply that the choices for the inclusion of these students would already bias the data towards non-swimmers who have relatively good dental relationships.

Breathing is characterized by two phases: the inspiratory phase where the thoracic cage expands due to the action of the inspiratory muscles, which creates an effect of absorption through which oxygen-rich air enters the lungs; then in the expiratory phase, the force of gravity and the relaxation of the inspiratory muscles allow the lungs to deflate, expelling the air that is contained in them, which is richer in carbon dioxide. In the case of forced expiration, there is also the necessary intervention of the expiratory muscles. During swimming, inspiration is always shorter but more powerful than expiration. The head and mouth positions also have key roles. During swimming, the athlete must learn how to breathe below the surface and to exhale under the water in a way that needs to be fluid (i.e. very close to natural), rhythmic (i.e. synchronized with body movements) and optimized (i.e. low energy consumption during swimming).

To assess the negative impact of mouth breathing on craniofacial development, Vargervik et al. [3] studied the effects of nasal airway obstruction in the rhesus monkey, and they demonstrated that mouth breathing can generate Class II malocclusion with crossbite, which will narrow and lengthen the palate and cause skeletal changes to the mandible.

When practised from childhood in particular, swimming teaches the swimmer to inspire and expire correctly: this is thus a real breathing exercise. Moreover, during swimming, there are frequent and prolonged periods of apnoea that force the swimmer to seal the lips and place the tongue correctly. Swallowing is also less frequent, to avoid swallowing water, which is always present in the oral cavity; in these moments, the tongue is placed at the rear of the oral cavity. The teeth are usually not in contact.

Indeed, numerous clinical observations have demonstrated the unequivocal contribution of the tongue in the development of jaw morphology [5, 8, 10, 15]. It is well known that only when there is a balance between the intraoral and extraoral muscles will there be harmonious dentoalveolar development and correct tooth eruption. Every muscular dysfunction, whether incorrect lingual posture (e.g. low tongue position, tongue thrusting, abnormal swallowing patterns) or perioral muscle contraction, can generate abnormal dentoalveolar changes [26–31]. Swimming is a symmetrical sport, and this ensures balanced and symmetrical development and muscle trophism throughout the body. Clinical experience shows that weak facial muscles in hyperdivergent patients, combined with oral breathing, generate open bite and crossbite, while hypodivergent patients are characterized by strong masseters, deep bite and retrusion of both the upper and lower alveolar bones [1, 15, 32–35].

In the present study, oral breathing, abnormal swallowing and incompetent lips were seen significantly more rarely with the swimmers than the non-swimmers. This confirms the assumptions on which this study is based, as well as the lower prevalence of the rates of crossbite, open bite and severe crowding that can be related to abnormal functional disorders [36].

Possible explanations that are linked to normal tongue function and posture, and to correct neuromuscular function, can be found primarily in the breathing methods used during swimming. These inspiration/expiration patterns follow according to well-known physiological models:

1. The cheeks are expanded by the air that is pushed out progressively but slowly through the closed lips, except for small openings at the sides. This promotes periosteum stimulation, which in turn induces new alveolar bone formation [14, 37, 38].
2. The air being pushed out increases the intraoral pressure, which becomes positive. Four valves in the orofacial area (i.e. nose, lips, soft palate, epiglottis) are involved in this process, and all of their correlated muscles are activated. This results in continuous exercise for correct mouth sealing and acquiring of the correct posture [14, 39].
3. The functional inspiratory/expiratory exercises involve the nose, mouth and pharynx, and these promote the correct development of the whole stomatognathic complex, particularly by repeating the same mechanism thousands of times, which ensures the maintenance of the physiological space in the oral cavity and airways. The establishment of the appropriate functional space [1, 14] is important, because it controls the epigenetic osteogenic growth processes that are encoded in the genomic sites (e.g. the capsular matrix, which through the muscles involved in tone and activity, determines the size and shape of the dental arches and the nasal cavity). Fränkel and Fränkel [14] stated that epigenetic control - both as an entity and in direction - influences endochondral and intramembranous growth. Yamada et al. [20] showed that in young rats swimming stimulates cartilage proliferation.
4. Tongue posture and activity: during swimming, the tongue rises from the palate, moving close to its rear, and it cannot therefore be interposed between the dental arches.
5. Positive pressure during expiration, together with correct tongue thrusting in the upper part of the palate: this might produce palatal expansion in childhood.
6. For the upper arch, all of these actions produce expansion, promoting a wider and more natural arch shape, and correct dental eruption. As the entire oral environment is positively affected, the lower arch will tend to have normal development, both opposing the upper arch by means of self-expansion, and having the same positive influences due to the expired air on the functional spaces.

The achievement of neuromuscular balance is supported by these aetiological assumptions, which appear to be confirmed by clinical and plaster model analysis of the dental arches of swimmers.

## Conclusions

The present study has shown that:

- there is significantly higher frequency of molar symmetry in swimmers versus non-swimmers;
- there is a significant increase in arch dimensions in swimmers versus non-swimmers, seen as +10% in the upper arch and +15% in the lower arch;
- there is significant crowding (greater than −3.5 mm) in the non-swimmers, in both the upper and lower arches;
- there is significantly lower prevalence rate of oral breathing and lip incompetence in swimmers versus non-swimmers.

The clinical findings of the present study appear to confirm our working hypotheses and to indicate that competitive swimming activities during childhood have positive effects on the development of the dental arches in the sagittal, vertical and transverse planes. These data once again confirm the importance of functional matrices in the development of the stomatognathic complex. Our future studies will evaluate morphometric and cephalometric modifications after competitive swimming activities, using lateral head films and/or cone beam computed tomography.
